# Mathematical and Computational Models for Pain: A Systematic Review

**DOI:** 10.1093/pm/pnab177

**Published:** 2021-05-29

**Authors:** Victoria Ashley Lang, Torbjörn Lundh, Max Ortiz-Catalan

**Affiliations:** 1 Center for Bionics and Pain Research, Sweden; 2 Department of Electrical Engineering, Chalmers University of Technology, Sweden; 3 Department of Mathematical Sciences, Chalmers University of Technology, Sweden; 4 Department of Mathematical Sciences, University of Gothenburg, Sweden; 5 Operational Area 3, Sahlgrenska University Hospital, Sweden; 6 Department of Orthopaedics, Institute of Clinical Sciences, Sahlgrenska Academy, University of Gothenburg, Sweden

**Keywords:** Pain Model, Mathematical Model, Computational Biology, Pain Mechanism

## Abstract

**Objective:**

There is no single prevailing theory of pain that explains its origin, qualities, and alleviation. Although many studies have investigated various molecular targets for pain management, few have attempted to examine the etiology or working mechanisms of pain through mathematical or computational model development. In this systematic review, we identified and classified mathematical and computational models for characterizing pain.

**Methods:**

The databases queried were *Science Direct* and *PubMed*, yielding 560 articles published prior to January 1st, 2020. After screening for inclusion of mathematical or computational models of pain, 31 articles were deemed relevant.

**Results:**

Most of the reviewed articles utilized classification algorithms to categorize pain and no-pain conditions. We found the literature heavily focused on the application of existing models or machine learning algorithms to identify the presence or absence of pain, rather than to explore features of pain that may be used for diagnostics and treatment.

**Conclusions:**

Although understudied, the development of mathematical models may augment the current understanding of pain by providing directions for testable hypotheses of its underlying mechanisms. Additional focus is needed on developing models that seek to understand the underlying mechanisms of pain, as this could potentially lead to major breakthroughs in its treatment.

## Introduction

Pain is a subjective experience mediated by a variety of physiological, psychological, and social factors. A personal painful experience may not be easy to communicate and may not be obvious to an observer, however, this lack of apparent objective evidence cannot negate the need for relief. Pain can exist without a physical stimulus, such as with neuropathic pains, and noxious stimuli do not always produce a painful experience, as seen in individuals with congenital insensitivity to pain [1]. This has led to a definition of pain that does not tie it to a physical stimulus. Instead, the International Association for Study of Pain (IASP) has recently redefined pain as “an unpleasant sensory and emotional experience associated with, or resembling that associated with, actual or potential tissue damage, or described in terms of such damage” [2].

There are many research efforts aiming to understand pain in order to develop pain relief strategies, particularly by using pharmaceutical methods [3]. Animal experiments dealing with pain have suggested possible pain pathways, regulatory systems, and modulatory schemes that may also exist in humans [4, 5]. Other attempts to alleviate pain include unconventional techniques such as hypnosis and acupuncture [6–9]. Decades of neuroimaging studies using human subjects have also vastly contributed to identifying regions involved in pain perception with respect to various pain conditions [10]. These studies have relied on statistical techniques, and more recently, machine learning, to make predictions on the relevance of brain regions to pain conditions [11]. Despite the breadth of data collected from brain imaging studies, statistical and machine learning approaches are limited by their inability to isolate biologically meaningful properties from others [12]. To date, an unconstrained method of studying the pain mechanism (i.e., free from uncontrollable variables and limited samples) has remained out of reach.

As with many other phenomena, pain can be studied at different scales. At the cellular level, hundreds of distinct changes had been identified as potential mediators of neuropathic pain, thus creating an equally large and challenging number of hypotheses yet to be tested [3]. A higher level of study would be that of neural network dynamics, which although encompasses cellular changes as reflected by neural firing behavior, does not require a complete understanding of the cellular changes themselves to utilize the resulting behavior in order to form working hypotheses. To this effect, mathematical and computational models of neural dynamics resulting in pain could be used to elucidate its etiology and serve as guidance for its treatment. Models are descriptions, abstract or material, that reflect or represent, and hence provide access to, selected parts of reality [13]. The development of a model requires 1) identification of the phenomenon to be examined, 2) determination of basic principles that drive the phenomenon, which are derived from the underlying theory, 3) validation of the model using experimental data, 4) iterative experimental work and model refinement with new data and mechanisms [13]. Once a model is validated, it can be used to better understand the subtleties of the observed phenomenon or to predict the behavior of the phenomenon under varying conditions. This model development process may generate new knowledge to improve an existing theory [13].

In order for a model to guide clinical treatment of pain, the model has to give insight into the pain condition by determining the relationships between factors or mechanisms that create the pain experience. A mechanistic model is designed to maximize understanding of the inner dynamics of the system through restricting the number of components for comprehensibility. This kind of model may reveal details about the pain mechanism that direct experimental efforts or clinical attention. The models proposed in Minamitani and Hagita [14] and Britton and Skevington [15] are bottom-up models that rely on fundamental physical concepts and are thus categorized as mechanistic models. In contrast, a predictive pain model is one that aims to identify pain parameters that are crucial to the presence or absence of pain. For example, the model presented by Keijsers et al. [16] is based on statistical methods and is categorized as a predictive model. While both models have their utility, at present, a deeper understanding of the internal pain dynamics may better direct efforts to treat pain. Hence, the focus should be on the explanatory power of a model, rather than only its predictive power. Ideally, a pain model should have both explanatory and predictive character in order to optimally facilitate theoretical development and clinical application. Mathematical and computational models of pain can be designed such that they may reveal undiscovered pain characteristics, explain clinical observations, or challenge current “truths” about pain mechanisms. To the best of our knowledge, this is the first review to focus on mathematical pain models. In this work, we examine the efforts made so far to understand pain via the development of said mathematical and computational models.

## Pain Theories

Until the second half of the 20th century, two main theories on pain were the *specificity theory* and the *pattern theory*. In 1662, Descartes suggested that pain was a product of neural processing and distinct from nociception [17]. He proposed that noxious stimuli were conveyed to the brain via hollow tubules, and a stimulus of adequate strength would evoke a painful sensation, while a weaker one would evoke a tingling or tickling sensation. Descartes’ theory of pain has since been elaborated and its details have culminated under the *specificity theory*, which suggests that stimulation of pain receptors produces nerve impulses that are transmitted to a pain center in the brain via pain-specific pathways. Under this theory, pain is considered an independent sensation, therefore requiring a separate sensory system for its perception—like for vision and hearing [17]. In contrast, *pattern theory* emerged through an effort to quantify sensation; the spatial-temporal pattern of impulses from the peripheral nerves encoded the type of sensation and its intensity. Weak and strong stimuli of the same modality could produce different patterns, thereby producing non-painful or painful experiences. This theory proposed that the central nervous system decoded these impulse patterns, but a sound explanation for this mechanism has not been made [17,18].

In 1965, Melzack and Wall [19] proposed the *gate control theory* of pain, in which spatial-temporal impulse patterns transmitted from peripheral afferent Aβ, Aδ, and C fibers are modulated at the spinal cord, and the modulated signals determine pain modality in the transmission cells in the dorsal horn, from which noxious information is projected towards the brain. The inclusion of the brain as an important component in pain perception was a major contribution of this theory, as stated by Melzack “never again, after 1965, could anyone try to explain pain exclusively in terms of peripheral factors” [20].

In the *gate control theory* of pain, nociceptive pain is mediated by unmyelinated C and Aδ fibers. C fibers transmit nociceptive signals from poorly localized noxious stimuli, while the slightly larger and thinly myelinated Aδ fibers mediate intense, acute pain. The largest nerve fibers—Aβ fibers—respond to touch, pressure, and tension. The *gate control theory* of pain suggests that Aβ fibers also play a role in pain, namely, in pain alleviation. This is exemplified in the short-term reduction of pain by applying light pressure to the painful region, like rubbing a stubbed toe, or applying pressure to the skin after removing a sticky bandage. Sufficient noxious input induces the firing of nociceptors, which can be achieved with appropriate mechanical, thermal, or electrical stimuli [21, 22].

No current theory is yet considered to account for all the intricacies of the experience of pain [17]. New ideas continue to emerge including other factors considered important to pain as a multidimensional experience, such as the neuromatrix theory [23], the mature organism model [24] or the dynamic pain connectome [25]. However, little guidance has been provided on how such ideas could be falsified, or experimentally verified.

Our group is particularly interested in phantom limb pain (PLP), a class of neuropathic pain commonly suffered after amputation of an extremity. Several hypotheses on the genesis of PLP were proposed over the last decades that are now challenged by clinical observations [26]. This motivated Ortiz-Catalan to conjecture the *stochastic entanglement hypothesis* for the genesis of PLP, in which pain and sensorimotor circuitry becomes pathologically linked to activate despite the lack of nociceptive input [26]. The somatosensory neural network is intrinsically linked to pain processing circuitry because all pain is embodied, that is, somatosensory processing provides the location in the body to a painful experience. The “entanglement” was hypothesized to initiate by incidental random firing of neurons belonging to the impaired sensorimotor network, unintentionally triggering neurons involved in pain perception. This and other ideas on the etiology and working mechanisms of pain, such as the more general *gate control theory* by Melzack [22] could be potentially studied using mathematical and computational models. The possible mechanisms that contribute to pain generation, perception, sensation, and alleviation are better illuminated by designing models that do not necessarily depend on datasets, but rather on known properties of components in the system. These models allow us to ask ourselves, “Do the properties of the modeled system necessarily result in the observations we make clinically, or are other outcomes possible?” Iteratively asking this question and making changes to the model would increase our understanding of the pain mechanism. The aim of this article is to identify and classify published efforts in this direction in order to inform future research.

## Methods

**Figure 1.**
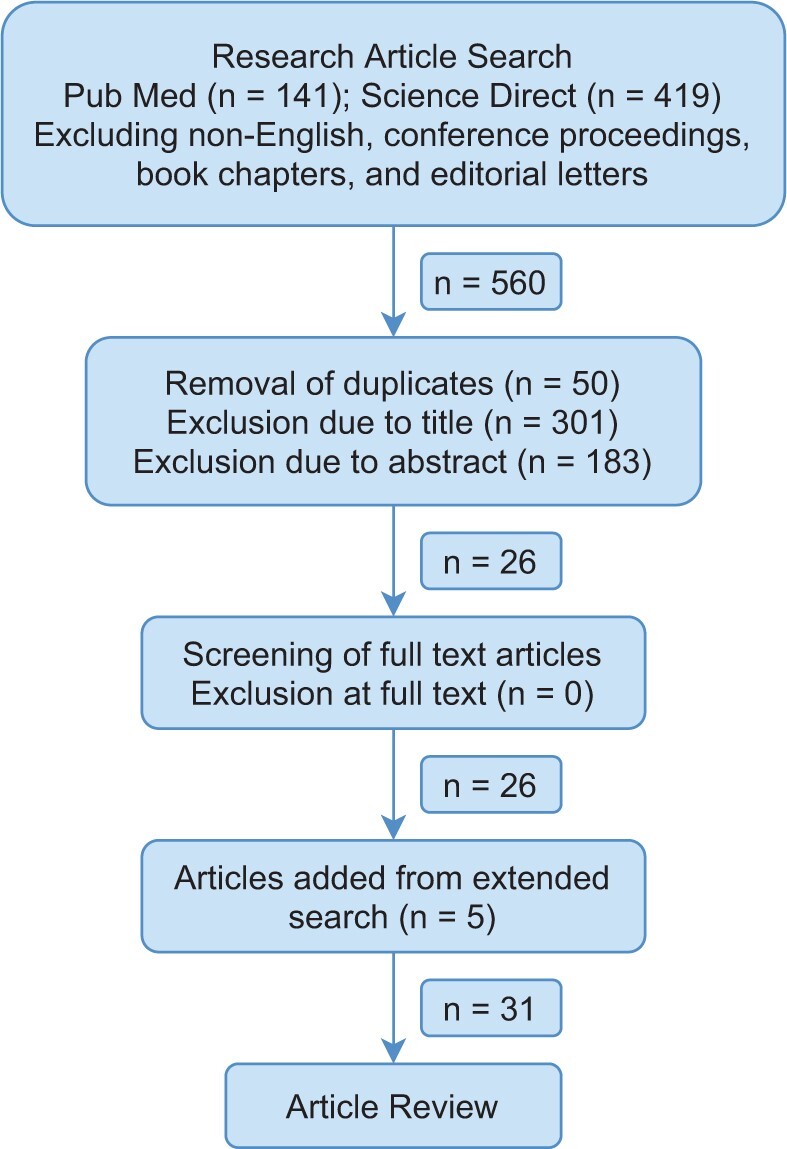
Schematic view of the methodology used for the systematic review. From the filtered search, the articles reviewed were required to contain a mathematical theory or computational approach to characterizing pain.

We performed a systematic literature review to identify and classify existing mathematical and computational models on pain in order to discern the current utility of these approaches. We searched the two databases: *Science Direct* and *PubMed*. Article title, key words, and abstracts were searched using the following search condition: (computational biology OR neural network OR mathematical model OR dynamical systems) AND pain AND (perception OR processing OR neuropathic OR chronic OR phantom limb). The inclusion criteria required the articles to contain a mathematical theory or computational approach to characterizing pain. Articles were not excluded based on model efficacy or predictive accuracy. We conducted no assessment of biases as this review is not concerned on the study outcomes but the models themselves. We considered journal articles published prior January 1st, 2020. Conference proceedings, book chapters, editorial letters, and non-English articles were excluded. The screening procedure is presented in detail in Figure 1.

**Figure 2.**
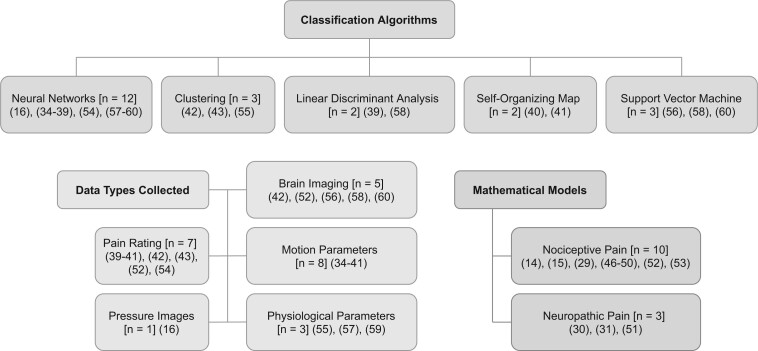
Articles sorted according to their classification algorithm, data collection method, or proposal of a mathematical model. Articles could belong to more than one category and subcategory.

The filtered search yielded 31 unique and relevant articles from 560 initially screened. Relevant articles discussed computational techniques for quantifying pain using clinical data and experiments, and computer simulations to replicate pain processing and perception. Articles removed due to title or abstract excluded content on modelling pain with mathematical or computational models. No articles were excluded after review at full-text. The peer-reviewed research articles were studied to identify the current mathematical and computational approaches to studying pain, and theories regarding the generation, qualities, and alleviation of pain. A breakdown of article types is given in Figure 2.

## Results

### Mathematical Models of Pain

In [13], on pp. 28–32, a model taxonomy is presented including conceptual, iconic, analogous, symbolic, phenomenological, and statistical models. This taxonomy was used in Table 1 where all articles found presenting mathematical models of pain were classified and described. Ten articles developed models for nociceptive pain and three for neuropathic pain.

**Table 1.** Literature on mathematical models of pain

Publication	Pain Type	Model Types* [13]	Summary
Minamitani and Hagita (1981) [14]	Nociceptive	Analogous/ Symbolic	The neural network model simulated the conduction mechanism of pain and touch sensations. Although only one directional ascending and descending pathway for pain sensation was represented, and no interaction from inhibition or facilitation was considered, the modalities of graded touch sensation and two different pain modalities were observed.
Britton and Skevington (1989) [15]	Nociceptive	Analogous/ Symbolic	Melzack's gate control theory of pain was translated into a mathematical model simulating acute pain for a single transmission unit. The partial differential equations were based on the Wilson-Cowan model for synaptically coupled neuronal networks.
Spitzer et al*.* (1995) [27]	Neuropathic (Phantom Limb)	Analogous/ Phenomenological	A self-organization feature map using Kohonen network was used to simulate the effects of amputation. The Kohonen network was trained on input patterns and subsequently deprived parts of the input patterns in order to simulate partial deafferentation. This led to reorganization driven by input noise, which represented noise generated by erratic firing of lacerated dorsal root ganglion sensory neurons.
Haeri et al. (2003) [28]	Nociceptive	Analogous/ Phenomenological	An artificial neural network to model the steady state behavior of pain mechanisms was developed using input patterns from small and large nerve fibers. For stimulation states corresponding to acute pain, a collection of basic patterns was used as features for the model. Given a novel pain stimulus, the prediction of pain was possible.
Xu et al. (2008) [29]	Nociceptive (Thermal)	Symbolic	Considering the biophysical and neural mechanisms of pain sensation, a mathematical model for quantifying skin thermal pain that included transduction, transmission, and perception was proposed. This model proposed that the intensity of thermal pain was related to the character of the noxious stimulus.
Cecchi et al. (2012) [30]	Nociceptive (Thermal)	Analogous/ Symbolic	Thermal pain perception was modelled as a dynamical system to be compared to reported pain ratings from intensity-varying thermal stimuli. Using a sparse regression method, pain ratings were predicted according to fMRI data and reported pain ratings.
Rho and Prescott (2012) [31]	Neuropathic	Conceptual/ Symbolic	A computational model was developed to simulate the onset of neuronal hyperexcitability from a normal spiking pattern. Parameters changes were sufficient to alter the normal spiking pattern to a repetitive one, enabling membrane potential oscillations, and bursting, suggesting that the three pathologies are related.
Boström et al. (2014) [32]	Neuropathic (Phantom Limb)	Conceptual/ Phenomenological	A computational model of phantom limb pain was developed based on the increase of spontaneous nociceptive firing. They proposed that the same underlying mechanism that results in ectopic spontaneous activity of deafferented nociceptive channels was responsible for phantom pain, maladaptive reorganization, and persistent representation.
Prince et al. (2014) [33]	Nociceptive	Analogous/ Symbolic	Britton and Skevington's acute pain model was replicated and expanded to verify the assumption that neighboring transmission units behave similarly. With sufficient increase in the number of transmission units input to the midbrain, transmission unit potential decreased, suggesting a saturation point in which transmission units may fail to fire despite neural fiber activation.
Tigerholm et al. (2014) [34]	Nociceptive	Analogous/ Symbolic	Axonal conduction velocity by activity differs between patients with neuropathic pain and those without, suggesting that this property may play a role in the development of neuropathies. A mathematical model of human cutaneous C-fibers was developed to investigate the activity-dependent changes of axonal spike conduction.
Dick et al. (2017) [35]	Nociceptive	Analogous/ Symbolic	By implementing a mathematical model of rat nociceptive neuronal membrane, a mechanism of ectopic bursting suppression in dorsal root ganglia neurons with comenic acid was proposed. The administration of comenic acid to the model reduced rhythmic discharge frequency due to a decrease in the effective charge transferring via sodium gate activation dynamics.
Crodelle et al. (2019) [36]	Nociceptive	Statistical/ Symbolic	A mathematical model of the dorsal horn neural circuit relying on firing rates and model parameters from experimental literature was developed to describe daily modulation of pain sensitivity. The inversion of daily rhythmicity of pain in neuropathic patients was proposed to be the result of dorsal horn circuitry dysregulation.
Dick (2020) [37]	Nociceptive	Analogous/ Symbolic	Bifurcation analysis was used to determine the relationship between the nociceptive neuron model and the antinociceptive effect that occurs during neuropathic pain suppression. The molecular mechanism of the bursting suppression was associated with the modification of the activation gating system of Nav1.8 channels by comenic acid, suggesting a possible molecular treatment for neuropathic pain.

* Conceptual models are the most basic of the model types. They are pedagogical and useful as foundations to more quantitative models. Analogous models borrow their structure from more well-known systems. Symbolic models are ordinarily described in mathematical language, i.e., symbols. Phenomenological models are symbolic in nature, but are often referred to as “black box models,” since their predictive power is the only priority. Statistical models are symbolic models where the mathematics is taken from probability theory. For additional details, see [13].

We found that the first quantitative analysis of nociceptive conduction was performed in 1981. Minamitani and Hagita proposed a mathematical model that generated a numerical description of nociceptive pain and touch sensations [14]. Based on findings in physiological and anatomical literature, including the gate control theory schematic from Melzack and Wall [19], the model simulated a one-directional ascending and descending pathway for pain sensation. Peripheral receptors, afferent Aβ, Aδ, and C fibers, and receptive neurons of the spinal cord, brain stem, thalamus, and the cerebral cortex were considered. To reduce complexity, interactions from lateral inhibition and facilitation were not included in the model. Even so, the model ended up with over 70 parameters. Whereas adaptation and conduction velocity of the fibers were considered, the fibers in each neural unit, consisting of the afferent fiber types, were prescribed with constant conduction velocity and firing threshold. The simulation was conducted with a single square-wave pulse and a periodic repetitive pulse applied to peripheral receptors. The activities of the neurons in the periphery and the upper brain were represented by Wilson-Cowan’s nonlinear differential equation. See the coupled pair of ordinary differential equations (ODE) [11] and [12] in [38], which considers continuous neuronal activity, and the distribution of peripheral receptors were described as Gaussian. This system was able to generate hysteresis and limit cycles; see also [39] for further details. The firing characteristics of the neurons were compared to physiological findings, where the results of the simulation and literature coincided satisfactorily. The modality of graded touch sensation, “fast stinging pain” mediated by small unmyelinated Aδ fibers, and “slow burning pain” mediated by unmyelinated C fibers were successfully simulated, despite the simplification of the model. This work suggested that this proposed neural network was useful to characterize different sensory modalities in pain.

**Figure 3.**
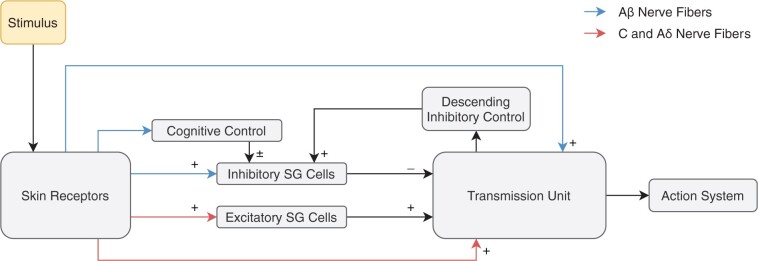
Melzack and Wall’s gate control theory of pain shown schematically. Compare with the original Figure 4 in [19] and the variants in Figure 1 in [15] and Figure 1 in [33]. Plus signs (+) denote excitation, and minus signs (−) denote inhibition. Cognitive control can be excitatory or inhibitory. SG = substantia gelatinosa cells in the dorsal horn of the spinal cord.

In 1989, Britton and Skevington translated the *gate control theory* of pain [19] into a mathematical model simulating acute pain for a single transmission unit, consisting of one Aβ fiber, one C fiber, one inhibitory neuron, and one excitatory neuron [15]. Figure 3 shows the gate control theory adapted schematically. They proposed a system of partial differential equations that exhibited all characteristics of acute pain described in the theory using the above mentioned Wilson-Cowan model of activity in a synaptically coupled neuronal network [38]. Furthermore, with some variation of parameters, they suggested that their model could demonstrate temporal pain qualities like throbbing and pulsing, and possibly expand the model’s validity to include neuropathy. This is quite remarkable given that the model is relatively minimalistic. See Equations (9) in [15] that consist of three coupled ODEs and only three unknown variables. This was utilized by the authors to prove analytic results about the uniqueness of a steady state solution. In 2004, this model was repeated and expanded, using more modern computational hardware and software, for the assumption that neighboring transmission units behave similarly [33]. The acute pain model was extended by increasing the number of transmission units communicating to the midbrain. Neighboring transmission units were found to behave similarly, but the transmission unit potential decreased when the number of transmission units increased sufficiently. This suggested a saturation point in which transmission units may fail to fire despite neural fiber activation [33].

In 2012, Rho and Prescott used relatively sophisticated tools and insights in non-linear dynamics to develop a computational model to simulate the onset of neuronal hyperexcitability from a normal spiking pattern [31]. Nerve injuries may cause various molecular changes, and any one change may singly cause neuropathy. The model demonstrated that the accumulation of small parameter changes was enough for 1) modifying the normal spiking pattern to a repetitive one, 2) enabling membrane potential oscillations, and 3) bursting. These changes suggested that all three pathological events are related. This study contrasts with other studies on neuropathic pain in that instead of identifying changes that result from pain, this model examined the combination of molecular changes that result in neuropathic excitability.

In 2014, Boström et al. developed a computational model of PLP, suggesting that phantom pain, maladaptive reorganization, and persistent representation are all symptoms of the same underlying mechanism that results in ectopic spontaneous activity of deafferented nociceptive channels [32]. They considered the somatosensory cortex to be dynamically self-organized, where spontaneous activity in the sensory system exists and is abnormally increased in regions affected by deafferentation. Following the *gate control theory’*s discussion on pain sensitization, they presume that a long-term decrease in input strength results in a similar change to the gating threshold. Their results suggest that amputation causes a threshold decrease, thereby allowing greater spontaneous activity in the sensory system. The assumptions required for this computational model consider nociception, but they excluded an explanation about a possible mechanism for pain perception or instantiation of a painful experience. Their model was based on PLP being the result of increased spontaneous nociceptive firing in the severed nerves, and thus initiated and maintained by the peripheral nervous system, which contradicts current views on PLP being maintained by central changes [40, 41].

### Pain Classification Algorithms

A considerable number of studies on pain involved pain questionnaires, experimental setups, and brain imaging. A small subset of these have used classification algorithms for identifying characteristics of pain from collected data. Table 2 lists, classifies, and describes the 18 articles found in this systematic review that relied on classification algorithms to differentiate between the presence or absence of pain.

**Table 2.** Literature on pain classification algorithms

Publication	Pain Type	Classification Algorithm	Input Data Type	Output Type	Summary
Gioftsos and Grieve (1996) [42]	CBP	ANN	Motion Parameters	Healthy Control, CBP, Fake CBP	Categorized chronic low back pain patients, fake low back pain patients, and healthy controls based on sit-to-stand maneuvers using an ANN and physiotherapist assessment. The ANN better detected abnormal movement patterns but was not necessarily better at diagnosing.
Oliver and Atsma (1996) [43]	CBP	ANN	Motion Parameters (sEMG)	Healthy Control, CBP	Categorized chronic low back pain patients and healthy controls using sEMG power spectra data collected from contraction tasks using an ANN.
Magnusson et al. (1998) [44]	CBP	ANN	Motion Parameters	Pre-Rehabilitation Motion Data, Post-Rehabilitation Motion Data	Identified chronic low back pain characteristics from trunk motion data in patients undergoing chronic low back pain rehabilitation.
Dickey et al. (2002) [45]	CBP	ANN, LDA	Motion Parameters, Pain Rating	Pain Response for Vertebral Motion	Chronic low back pain motion, intravertebral deformation, and pain were assessed with LDA and an ANN. The ANN showed a strong relationship between observed and predicted pain due to the nonlinear relationship between vertebral motion parameters and pain.
Liszka and Martin (2002) [46]	CBP	SOM	Motion Parameters, Pain Rating	Functional Status (SF-36 Score)	Categorized pain and activity levels through functional status derived from the SF-36 questionnaire from patients with acute back pain and chronic back pain. The correlation coefficient between the true and predicted SF-36 scores for mental and general health were significant with the inclusion of activity and pain data.
Liszka and Martin (2005) [47]	CBP	SOM	Motion Parameters, Pain Rating		Investigated the relationship between daytime chronic back pain levels and sleep activity using a SOM neural network. Results showed that daytime pain levels and sleep activity were not correlated, however, daytime pain variance was correlated with sleep activity levels and patterns.
Balaban et al. (2005) [48]	Nociceptive (Chemical)	Cluster Analysis	Pain Rating	3 Capsaicin Response Phenotypes	Identified 3 response phenotypes (level detection, change detection, and cumulative irritation) to 2 time-varying capsaicin administration paradigms and proposed a method of classifying human pain responses by temporal pattern, rather than by threshold or magnitude of response.
Behrman et al. (2007) [49]	Neuropathic	ANN	Pain Rating	Neuropathic Pain, Non-neuropathic Pain	Compared an ANN to traditional scoring systems for differentiating neuropathic pain and non-neuropathic pain patients using responses from a neuropathic pain questionnaire. Argued that nonlinearities within data are insignificant since both classification methods achieved similar results.
Cannistraci et al. (2010) [50]	Neuropathic	Cluster Analysis	Cerebrospinal Fluid	Neuropathic Pain, Non-neuropathic Pain	Proposed *Minimum Curvilinearity* for dimension reduction and clustering for the classification of 2 D electrophoresis gel images derived from proteomic cerebrospinal fluid profiles of peripheral neuropathic patients and ALS patients unaffected by neuropathic pain. This method improved classification accuracy of small nonlinear datasets.
Brodersen et al. (2012) [51]	Nociceptive (Thermal)	SVM	Brain Imaging (fMRI)		Investigated the predictive ability of fMRI data for decoding painful stimuli using multivariate analysis on different spatial scales (single voxels, individual anatomical regions, combinations of regions, or whole-brain activity).
Keijsers et al. [16]	Nociceptive (Mechanical)	ANN	Pressure Images	Healthy Control, Forefoot Pain	Identified differences in plantar pressure patterns in people with and without forefoot pain.
Atlas et al. (2014) [52]	Nociceptive (Thermal)	Cluster Analysis	Brain Imaging (fMRI), Pain Rating (VAS)		Identified brain mediators of pain induced by thermal stimuli using multi-level mediation analysis. Cluster analysis showed that the mediators belonged to several distinct functional networks with complementary roles in pain genesis. The identified networks did not necessarily respond to noxious input and predict pain, indicating various brain regions contribute to the pain.
Ozkan et al. (2015) [53]	Fibromyalgia	ANN	SSR, Various Physiological Tests	Healthy Control, Fibromyalgia Pain	Demonstrated that the inclusion of SSR parameters as a feature in a fibromyalgia ANN model increases classification accuracy from 96.51% to 97.67%. Argued that SSR parameters could be used as new auxillary diagnostic factors for fibromyalgia.
Caza et al. (2016) [54]	CBP	ANN	Motion Parameters (sEMG)	Healthy Control, CBP	Categorized chronic low back pain patients and healthy controls from sEMG data collected from a muscle endurance task. A surrogate analysis of the data scored each channel on the sEMG sensory array based on the fractal dimension, showing nonlinearity. The most nonlinear values were used as signal characteristics for the ANN model.
Hu et al. (2018) [55]	CBP	ANN	Motion Parameters	Healthy Control, CBP	Identified low back pain patients using static balance control performance data during static standing tasks.
Vuckovic et al. (2018) [56]	Neuropathic (Spinal Cord Injury)	ANN, LDA, SVM	Brain Imaging (EEG)	Healthy Control, At-Risk Group	Classified spinal cord injured patients at risk of developing neuropathic pain by comparing them to patients who had already developed pain and healthy controls.
Henssen et al. (2019) [57]	Chronic Intractable	ANN	Patient History, Experimental Variables		Identified predictive variables that influence the outcome of implantable motor cortex stimulation for intractable pain.
Santana et al. (2019) [58]	CBP, Fibromyalgia	ANN, SVM	Brain Imaging (fMRI)		Resting-state fMRI data from chronic pain patients and healthy controls were collected to assess the accuracy of different machine learning models for classification of chronic pain.

Twelve of the 18 used neural networks, and 6 of these were applied to identify chronic low back pain using parameters acquired from surface electromyography (sEMG), kinematic data, images, or video. Classification accuracies measured 80% or higher, indicating neural networks could be complementary to physiotherapist assessments [42–45,54, 55]. Two additional articles utilized self-organizing maps to categorize patients with and without chronic low back pain from activity levels and pain questionnaires [46, 47].

In another effort to autonomously classify data, Atlas et al. used cluster analysis with fMRI, demonstrating some brain regions responded to the intensity of a thermal stimulus but did not predict pain [52]. Other brain regions did not respond to noxious heat input but did predict pain. Balaban et al. used fuzzy C-means cluster analysis to identify three response phenotypes to two time-varying oral capsaicin administration paradigms in order to examine individual pain differences [48]. This work proposed classification of human pain responses by temporal pattern, rather than by threshold or magnitude of response.

## Discussion

Owing to its complexity, understanding pain in all its varieties has proven challenging, and therefore many efforts are made toward understanding factors that contribute to specific pain conditions. Often these efforts require experimental laboratory work or clinical studies which can have long reporting times and are unique to the molecules explored or subject data collected. Mathematical models are advantageous in that they do not require data acquisition until validation, thereby permitting the development of several models and subsequently corroborating each with experimental data simultaneously. Furthermore, mathematical models can be designed to examine specific or general phenomena. Despite these apparent benefits, the publications we found on mathematical modelling of pain averaged 17.8 citations (±14.1) by December 31^st^, 2020, which is an indication that mathematical modelling has not been a tool widely employed nor accepted in the study of pain. This is notwithstanding its proven utility in helping to understand complex biological processes, such as in exploring the neural dynamics of vision and in predicting the spread of infectious diseases [59, 60].

A possible explanation for the lack of mathematical models on pain is the lack of appropriate data to test them. There are many molecular candidates for targeted pain relief [3], but their interactions and dynamics are difficult to inject into a mathematical theory for pain without creating a model unique to a single set of molecules and their conditions. There is an ever-growing number of parameters to consider, and the complexity of developing a robust model that does not oversimplify the pain experience is a mounting barrier; see for example [13]. In Minamitani and Hagita’s model [14], even without considering lateral inhibition and facilitation, their model utilizes over 70 parameters. The sheer number of parameters complicates model construction such that it is a deterrent to replications and expansions. Furthermore, the psychological factors that affect the pain experience are difficult to include in mechanistic models due to the required simulation of other higher-order brain processes like memory, attention, and learning. Even with concurrent simulation of related higher-order brain functions, the number of parameters to model would considerably increase, thereby significantly increasing the complexity of the model and reducing its comprehensibility. At this point, it is unclear which biomarkers are present in all instances of pain, hence the difficulty in creating an all-inclusive model.

We found that the few mathematical models that tackled the root of pain generation often relied on simplified representations of neural mechanism and missed the mark of mimicking the variety of pain types. In Britton and Skevington’s 1989 mathematical model of the *gate control theory*, acute pain was successfully simulated and further analysis suggested an explanation for temporal qualities of pain, a phenomenon previously unexplained by this theory [15]. However elegantly constructed from a set of three coupled ODEs, the parameters and functions were only generally described and essentially not based on experimental measurements. The possibility of modifying the model to also simulate chronic pain via the inclusion of a plasticity scheme was surprisingly never realized in any future work [28,33], despite its tangibility. Efforts towards a general understanding of pain and its origin diminished in more recent decades, in favor of models for specific pain conditions.

Recent models have examined specific aspects of pain, such as daily variance of intensity, nociceptor response to chemical or thermal stimuli, and PLP [29,32,35–37]. The limitation of these models lies in the lack of generalizability. The computational model of PLP developed by Boström et al. [32] is one that claims that spontaneous activity in the somatosensory cortex is abnormally increased in regions affected by deafferentation, in which PLP arises. In line with mechanisms of pain sensitization from the *gate control theory*, they assume that amputation puts no constraint on nociceptive input due to the lack of sensory input, leading to a decrease in gating threshold and thereby an increase in spontaneous activity. Self-organization of the somatosensory cortex would maladapt to this sustained absence of sensory information and result in PLP [32]. Although nociception is considered in this model, no pain mechanism is discussed that supports maintenance of firing at the severed nerves. Despite the complexity of this model, the results were expected given that their model was constructed using rather strong assumptions on the underlying PLP process and well-known but complex phenomenologically model parts, such as Kohonen maps (also known as self-organizing maps, SOM).

Articles discussing pain classification algorithms averaged 24.7 citations (SD = 28.0). Most of this literature focused on categorizing behavioral and brain imaging data to identify the presence of pain. The primary focus of these articles was on developing more effective methods of identifying characteristics and patterns of pain in order to give more accurate predictions. The use of artificial neural networks was successful for identifying patients with chronic low back pain using motion parameters and sEMG, suggesting its diagnostic power. However, none of these articles attempted to differentiate no pain, acute pain, and chronic pain due to limitations set on the scope of the experiments. In studies comparing clinician and algorithmic predictive successes, there were missed opportunities to scrutinize examples in which the clinician and algorithm disagreed. This error analysis could have been used to improve the algorithms and to augment the clinician’s knowledge in diagnosing pain conditions. Lastly, the reliance on collecting suitable data types in sufficient amounts is detrimental when relying on these classification methods. Despite these challenges, computational models are invaluable tools for examining the full spectrum of conditions because combinations of parameters can be studied faster than with clinical studies, which cannot test exhaustively.

Finally, we encourage researchers to provide experimental procedures to falsify their proposed hypothesis, such as those illustrated by Devor on molecular mechanisms [3] and Ortiz-Catalan on PLP [26].

## Notes

Funding sources: This research was supported by the Promobilia Foundation, the IngaBritt and Arne Lundbergs Foundation, Swedish Research Council (Vetenskapsrådet), and the European Pain Federation–Grünenthal–Research Grant (E-G-G).
